# Bis(μ-4-nitro-2-{[2-(oxidometh­yl)phen­yl]imino­meth­yl}phenolato)bis­[chlorido(dimethyl sulfoxide)­iron(III)] dimethyl sulfoxide disolvate

**DOI:** 10.1107/S1600536812017424

**Published:** 2012-04-25

**Authors:** Eduard N. Chygorin, Julia A. Rusanova, Roman I. Zubatyuk, Oleg V. Shishkin

**Affiliations:** a Taras Shevchenko National University of Kyiv, 64, Volodymyrs’ka St., 01601 Kyiv, Ukraine; b STC Institute for Single Crystals, National Academy of Sciences of Ukraine, Lenina ave. 60, Kharkov 61001, Ukraine

## Abstract

In the centrosymmetric dimeric title complex, [Fe_2_(C_14_H_10_N_2_O_4_)_2_Cl_2_(C_2_H_6_OS)_2_]·2C_2_H_6_OS, two {Fe(*L*)Cl(DMSO)} units (*L* is the tridentate ligand 4-nitro-2-{[2-(oxidometh­yl)phen­yl]imino­meth­yl}phenolate; DMSO is dimethyl sulfoxide) are bridged by two O atoms, with an Fe⋯Fe separation of 3.1838 (8) Å. The coordination polyhedron of the Fe^III^ atoms can be described as distorted octa­hedral, with four Fe—O, one Fe—N and one Fe—Cl coordination bonds. The *L* ligand is not planar, the dihedral angle between the 2-(oxidometh­yl)phen­yl]imino and 4-nitro-2-(imino­meth­yl)phenolate planes being 48.54 (9)°. The solvent DMSO molecule is disordered over two orientations with equal occupancy.

## Related literature

For background to direct synthesis, see: Vassilyeva *et al.* (1997 ▶); Babich & Kokozay (1997 ▶); Kovbasyuk *et al.* (1997 ▶, 1998 ▶); Makhankova *et al.* (2002 ▶); Vinogradova *et al.* (2002 ▶); Pryma *et al.* (2003 ▶); Nesterov *et al.* (2004 ▶). For the structures of related complexes, see: Elmali *et al.* (2000 ▶); Chen *et al.* (2001 ▶); Koikawa *et al.* (2004 ▶); Madhu *et al.* (2005 ▶); Malassa *et al.* (2006 ▶).
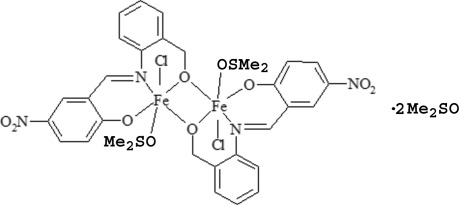


## Experimental

### 

#### Crystal data

[Fe_2_(C_14_H_10_N_2_O_4_)_2_Cl_2_(C_2_H_6_OS)_2_]·2C_2_H_6_OS
*M* *_r_* = 1035.59
Monoclinic, 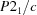

*a* = 13.5003 (10) Å
*b* = 10.2566 (6) Å
*c* = 16.7453 (12) Å
β = 97.027 (6)°
*V* = 2301.3 (3) Å^3^
*Z* = 2
Mo *K*α radiation
μ = 0.99 mm^−1^
*T* = 293 K
0.35 × 0.05 × 0.04 mm

#### Data collection

Oxford Diffraction Xcalibur Sapphire3 diffractometer
Absorption correction: multi-scan (*CrysAlis PRO*; Oxford Diffraction, 2010 ▶). *T*_min_ = 0.917, *T*_max_ = 0.961
20394 measured reflections
5457 independent reflections
3448 reflections with *I* > 2σ(*I*)
*R*_int_ = 0.050

#### Refinement

*R*[*F* ^2^ > 2σ(*F* ^2^)] = 0.059
*wR*(*F* ^2^) = 0.160
*S* = 1.05
5457 reflections
282 parameters
H-atom parameters constrained
Δρ_max_ = 0.43 e Å^−3^
Δρ_min_ = −0.30 e Å^−3^

### 

Data collection: *CrysAlis PRO* (Oxford Diffraction, 2010 ▶); cell refinement: *CrysAlis PRO*; data reduction: *CrysAlis PRO*; program(s) used to solve structure: *SHELXTL* (Sheldrick, 2008 ▶); program(s) used to refine structure: *SHELXTL*; molecular graphics: *SHELXTL*; software used to prepare material for publication: *publCIF* (Westrip, 2010 ▶).

## Supplementary Material

Crystal structure: contains datablock(s) I, global. DOI: 10.1107/S1600536812017424/br2198sup1.cif

Structure factors: contains datablock(s) I. DOI: 10.1107/S1600536812017424/br2198Isup2.hkl

Additional supplementary materials:  crystallographic information; 3D view; checkCIF report

## Figures and Tables

**Fig. 1.**
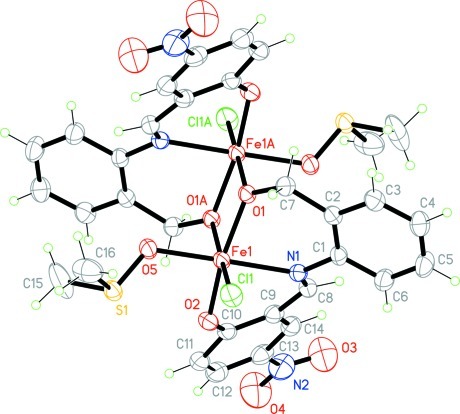
Molecular view of the title compound. Displacement ellipsoids are drawn at the 30% probability level.

## supplementary crystallographic information

### Comment

This work is a continuation of our research in the field of direct synthesis of coordination compounds, which employs metal powders or metal oxides as starting materials and has been proved to be an efficient route to obtain homo- and heterometallic complexes (Vassilyeva *et al.*, (1997); Babich *et al.*, (1997); Kovbasyuk *et al.*, (1997, 1998); Makhankova *et al.*, (2002); Vinogradova *et al.*, (2002); Pryma *et al.*, (2003); Nesterov *et al.*, (2004)).

The title compound, [Fe(C_14_H_10_N_2_O_3_)(DMSO)Cl]_2_.2DMSO was obtained unintentionally as the product of an attempted synthesis of a Cu/Fe mixed-metal complex using zerovalent copper, iron(II) chloride tetrahydrate, 5-nitro-salycilic aldehyde, 2-aminobenzylalcohol, triethylamine in dimethyl sulfoxide. It consists of dimer [Fe(C_14_H_10_N_2_O_3_)(DMSO)Cl]_2_ and two disordered solvent (DMSO) molecules which are filled voids in crystal packing.

The crystal structure of the title complex without solvent molecules which are omited for clarity is shown in Fig. 1. In contrast to analogous complex [FeCl(C_14_H_11_NO_2_)]_2_ (Koikawa *et al.*, (2004)) with 5 coordinated Fe atom, in the title complex there is an additional bond Fe –O from the coordinated DMSO molecule, which leads to significant changes in geometry of the iron atom coordination environment – increasing of coordination number to 6 and to a redistribution of bond lengths. Almost unchangeable remains only Fe –O distance with alcohol oxygen atom. The Fe···Fe distances in title compound as well as other bond distances and angles are comparable to the corresponding distances in closely related compounds (Elmali *et al.*, (2000); Chen *et al.*, (2001); Koikawa *et al.*, (2004).; Madhu *et al.*, (2005); Malassa *et al.*, (2006)).

### Experimental

The title compound was prepared by direct synthesis by addition of the zero valent copper powder 0,079 g (1,25 mmol) and FeCl_2_^.^ 4H_2_O 0,248 g (1,25 mmol) to the previously mixed within about 10 min at 323–333 K (until the yellow color) mixture of the 5-nitro-salycilic aldehyde 0,418 g (2,5 mmol), 2-aminobenzylalcohol 0,308 g (2,5 mmol), DMSO 25 ml and triethylamine 0,350 ml (2,5 mmol) and stirred magnetically for 4.5 h till complete dissolution of copper powder was observed. Dark red crystals suitable for X-ray analysis precipitated within two months by adding of Pr^í^—OH and diethyl ether to the dark red solution. They were collected by filter-suction, washed with dry Pr^í^—OH and finally dried *in vacuo* at room temperature (yield: 0.2 g).

### Refinement

Solvate DMSO molecule is disordered over two position A and B only for sulfur atom with equal multiplicity. All H atoms were placed at calculated positions and refinement as a "riding" model.

### Crystal data

**Table:**

[Fe_2_(C_14_H_10_N_2_O_4_)_2_Cl_2_(C_2_H_6_OS)_2_]·2C_2_H_6_OS	*F*(000) = 1068
*M**_r_* = 1035.59	*D*_x_ = 1.495 Mg m^−^^3^
Monoclinic, *P*2_1_/*c*	Mo *K*α radiation, λ = 0.7107 Å
Hall symbol: -P 2ybc	Cell parameters from 4779 reflections
*a* = 13.5003 (10) Å	θ = 2.9–28.6°
*b* = 10.2566 (6) Å	µ = 0.99 mm^−^^1^
*c* = 16.7453 (12) Å	*T* = 293 K
β = 97.027 (6)°	Prism, dark-red
*V* = 2301.3 (3) Å^3^	0.35 × 0.05 × 0.04 mm
*Z* = 2	

### Data collection

**Table:**

Oxford Diffraction Xcalibur Sapphire3 diffractometer	5457 independent reflections
Radiation source: Enhance (Mo) X-ray Source	3448 reflections with *I* > 2σ(*I*)
Graphite monochromator	*R*_int_ = 0.050
Detector resolution: 16.1827 pixels mm^-1^	θ_max_ = 28.7°, θ_min_ = 2.9°
ω scans	*h* = −18→17
Absorption correction: multi-scan *CrysAlis PRO*, Oxford Diffraction (2010).	*k* = −12→13
*T*_min_ = 0.917, *T*_max_ = 0.961	*l* = −22→20
20394 measured reflections	

### Refinement

**Table:**

Refinement on *F*^2^	Primary atom site location: structure-invariant direct methods
Least-squares matrix: full	Secondary atom site location: difference Fourier map
*R*[*F*^2^ > 2σ(*F*^2^)] = 0.059	Hydrogen site location: inferred from neighbouring sites
*wR*(*F*^2^) = 0.160	H-atom parameters constrained
*S* = 1.05	*w* = 1/[σ^2^(*F*_o_^2^) + (0.0648*P*)^2^ + 2.0858*P*] where *P* = (*F*_o_^2^ + 2*F*_c_^2^)/3
5457 reflections	(Δ/σ)_max_ < 0.001
282 parameters	Δρ_max_ = 0.43 e Å^−^^3^
0 restraints	Δρ_min_ = −0.30 e Å^−^^3^

### Special details

**Table:**

Geometry. All e.s.d.'s (except the e.s.d. in the dihedral angle between two l.s. planes) are estimated using the full covariance matrix. The cell e.s.d.'s are taken into account individually in the estimation of e.s.d.'s in distances, angles and torsion angles; correlations between e.s.d.'s in cell parameters are only used when they are defined by crystal symmetry. An approximate (isotropic) treatment of cell e.s.d.'s is used for estimating e.s.d.'s involving l.s. planes.
Refinement. Refinement of *F*^2^ against ALL reflections. The weighted *R*-factor *wR* and goodness of fit *S* are based on *F*^2^, conventional *R*-factors *R* are based on *F*, with *F* set to zero for negative *F*^2^. The threshold expression of *F*^2^ > σ(*F*^2^) is used only for calculating *R*-factors(gt) *etc*. and is not relevant to the choice of reflections for refinement. *R*-factors based on *F*^2^ are statistically about twice as large as those based on *F*, and *R*- factors based on ALL data will be even larger.

### Fractional atomic coordinates and isotropic or equivalent isotropic displacement parameters (Å^2^)

**Table:**

	*x*	*y*	*z*	*U*_iso_*/*U*_eq_	Occ. (<1)
Fe1	0.54824 (4)	0.35880 (5)	0.01293 (3)	0.04140 (18)	
Cl1	0.56904 (9)	0.20842 (10)	0.11735 (7)	0.0625 (3)	
S1	0.41820 (8)	0.12129 (9)	−0.05553 (7)	0.0503 (3)	
S2A	0.9728 (3)	0.8274 (3)	−0.0026 (3)	0.1070 (13)	0.50
S2B	0.9931 (3)	0.7273 (4)	0.0176 (3)	0.1014 (11)	0.50
O1	0.48948 (18)	0.4976 (2)	0.07173 (14)	0.0400 (6)	
O2	0.6276 (2)	0.2709 (3)	−0.06064 (17)	0.0525 (7)	
O3	0.9361 (4)	0.6035 (5)	−0.2201 (3)	0.1141 (16)	
O4	0.8945 (4)	0.4653 (6)	−0.3122 (3)	0.1294 (18)	
O5	0.4218 (2)	0.2671 (2)	−0.03552 (17)	0.0511 (7)	
O6	0.8873 (4)	0.7636 (6)	−0.0133 (4)	0.149 (2)	
N1	0.6840 (2)	0.4594 (3)	0.05581 (19)	0.0418 (7)	
N2	0.8868 (4)	0.5106 (6)	−0.2461 (3)	0.0842 (14)	
C1	0.7040 (3)	0.5091 (4)	0.1360 (2)	0.0436 (9)	
C2	0.6269 (3)	0.5362 (3)	0.1821 (2)	0.0430 (9)	
C3	0.6529 (4)	0.5857 (4)	0.2587 (2)	0.0544 (11)	
H3	0.6024	0.6068	0.2896	0.065*	
C4	0.7499 (4)	0.6049 (5)	0.2908 (3)	0.0647 (13)	
H4	0.7644	0.6391	0.3423	0.078*	
C5	0.8258 (4)	0.5735 (5)	0.2467 (3)	0.0698 (14)	
H5	0.8921	0.5849	0.2682	0.084*	
C6	0.8024 (3)	0.5250 (5)	0.1704 (3)	0.0590 (11)	
H6	0.8537	0.5020	0.1409	0.071*	
C7	0.5167 (3)	0.5185 (4)	0.1551 (2)	0.0460 (9)	
H7A	0.4932	0.4450	0.1840	0.055*	
H7B	0.4819	0.5954	0.1708	0.055*	
C8	0.7399 (3)	0.4989 (4)	0.0034 (2)	0.0482 (10)	
H8	0.7824	0.5686	0.0179	0.058*	
C9	0.7420 (3)	0.4444 (4)	−0.0756 (2)	0.0475 (9)	
C10	0.6896 (3)	0.3290 (4)	−0.1021 (3)	0.0491 (10)	
C11	0.7062 (3)	0.2774 (5)	−0.1774 (3)	0.0587 (12)	
H11	0.6737	0.2010	−0.1954	0.070*	
C12	0.7681 (4)	0.3357 (5)	−0.2244 (3)	0.0669 (14)	
H12	0.7770	0.3003	−0.2741	0.080*	
C13	0.8176 (3)	0.4479 (5)	−0.1977 (3)	0.0605 (12)	
C14	0.8059 (3)	0.5023 (5)	−0.1244 (3)	0.0563 (11)	
H14	0.8406	0.5776	−0.1074	0.068*	
C15	0.3493 (7)	0.1186 (5)	−0.1514 (4)	0.128 (3)	
H15A	0.2846	0.1559	−0.1486	0.192*	
H15B	0.3419	0.0302	−0.1699	0.192*	
H15C	0.3836	0.1683	−0.1880	0.192*	
C16	0.3284 (5)	0.0564 (5)	0.0007 (4)	0.0949 (19)	
H16A	0.3556	0.0515	0.0564	0.142*	
H16B	0.3099	−0.0294	−0.0187	0.142*	
H16C	0.2705	0.1116	−0.0047	0.142*	
C17	1.0304 (7)	0.8170 (11)	0.0944 (5)	0.185 (5)	
H17A	0.9879	0.8539	0.1304	0.278*	0.50
H17B	1.0924	0.8640	0.0990	0.278*	0.50
H17C	1.0432	0.7271	0.1081	0.278*	0.50
H17D	1.0274	0.9071	0.0788	0.278*	0.50
H17E	1.0979	0.7946	0.1145	0.278*	0.50
H17F	0.9882	0.8027	0.1357	0.278*	0.50
C18	1.0679 (6)	0.7618 (11)	−0.0498 (5)	0.161 (4)	
H18A	1.0807	0.6741	−0.0313	0.242*	0.50
H18B	1.1271	0.8134	−0.0374	0.242*	0.50
H18C	1.0487	0.7613	−0.1069	0.242*	0.50
H18D	1.0479	0.7132	−0.0981	0.242*	0.50
H18E	1.1351	0.7390	−0.0291	0.242*	0.50
H18F	1.0643	0.8534	−0.0616	0.242*	0.50

### Atomic displacement parameters (Å^2^)

**Table:**

	*U*^11^	*U*^22^	*U*^33^	*U*^12^	*U*^13^	*U*^23^
Fe1	0.0469 (3)	0.0338 (3)	0.0417 (3)	0.0014 (2)	−0.0023 (2)	−0.0014 (2)
Cl1	0.0832 (8)	0.0466 (6)	0.0540 (7)	0.0007 (5)	−0.0065 (6)	0.0103 (5)
S1	0.0594 (6)	0.0376 (5)	0.0516 (6)	−0.0015 (4)	−0.0028 (5)	−0.0012 (4)
S2A	0.091 (2)	0.0677 (19)	0.154 (4)	−0.0049 (17)	−0.022 (2)	0.008 (2)
S2B	0.095 (3)	0.086 (2)	0.122 (3)	0.0097 (19)	0.007 (2)	0.003 (2)
O1	0.0461 (15)	0.0377 (13)	0.0346 (14)	0.0014 (11)	−0.0013 (11)	0.0006 (10)
O2	0.0589 (18)	0.0421 (15)	0.0556 (18)	0.0060 (13)	0.0035 (15)	−0.0089 (13)
O3	0.120 (4)	0.138 (4)	0.093 (3)	−0.038 (3)	0.048 (3)	−0.010 (3)
O4	0.135 (4)	0.185 (5)	0.080 (3)	−0.030 (4)	0.061 (3)	−0.033 (3)
O5	0.0547 (17)	0.0385 (14)	0.0566 (17)	−0.0014 (12)	−0.0075 (14)	−0.0036 (12)
O6	0.106 (4)	0.166 (5)	0.171 (6)	−0.007 (4)	−0.004 (4)	−0.035 (4)
N1	0.0431 (18)	0.0406 (16)	0.0410 (18)	0.0047 (14)	0.0018 (15)	−0.0025 (14)
N2	0.075 (3)	0.116 (4)	0.064 (3)	0.002 (3)	0.021 (2)	−0.007 (3)
C1	0.049 (2)	0.041 (2)	0.039 (2)	−0.0025 (17)	−0.0016 (18)	−0.0005 (16)
C2	0.055 (2)	0.0372 (19)	0.035 (2)	0.0019 (17)	−0.0027 (18)	0.0032 (16)
C3	0.067 (3)	0.056 (2)	0.039 (2)	0.004 (2)	0.002 (2)	0.0011 (19)
C4	0.075 (3)	0.079 (3)	0.037 (2)	−0.005 (3)	−0.006 (2)	−0.005 (2)
C5	0.057 (3)	0.094 (4)	0.054 (3)	−0.007 (3)	−0.010 (2)	−0.007 (3)
C6	0.051 (3)	0.071 (3)	0.053 (3)	0.000 (2)	0.000 (2)	−0.007 (2)
C7	0.052 (2)	0.052 (2)	0.034 (2)	0.0045 (19)	0.0034 (17)	0.0009 (17)
C8	0.048 (2)	0.044 (2)	0.050 (2)	0.0010 (18)	−0.001 (2)	−0.0050 (18)
C9	0.047 (2)	0.054 (2)	0.041 (2)	0.0083 (18)	0.0032 (18)	−0.0002 (19)
C10	0.050 (2)	0.048 (2)	0.047 (2)	0.0150 (18)	−0.0036 (19)	−0.0059 (18)
C11	0.056 (3)	0.065 (3)	0.053 (3)	0.012 (2)	0.000 (2)	−0.016 (2)
C12	0.062 (3)	0.092 (4)	0.046 (3)	0.021 (3)	0.001 (2)	−0.018 (3)
C13	0.049 (3)	0.085 (3)	0.049 (3)	0.014 (2)	0.010 (2)	0.000 (2)
C14	0.049 (2)	0.065 (3)	0.053 (3)	0.007 (2)	0.001 (2)	−0.005 (2)
C15	0.219 (9)	0.055 (3)	0.088 (5)	−0.010 (4)	−0.071 (5)	−0.006 (3)
C16	0.094 (4)	0.061 (3)	0.139 (6)	−0.004 (3)	0.050 (4)	0.003 (3)
C17	0.143 (8)	0.269 (12)	0.129 (7)	0.099 (8)	−0.044 (6)	−0.062 (8)
C18	0.126 (7)	0.210 (10)	0.151 (8)	0.053 (7)	0.030 (6)	0.036 (8)

### Geometric parameters (Å, º)

**Table:**

Fe1—Cl1	2.3228 (11)	C6—H6	0.9300
Fe1—O1	1.954 (2)	C7—H7A	0.9700
Fe1—O1^i^	2.064 (2)	C7—H7B	0.9700
Fe1—O2	1.949 (3)	C8—H8	0.9300
Fe1—O5	2.030 (3)	C8—C9	1.440 (6)
Fe1—N1	2.149 (3)	C9—C10	1.422 (6)
S1—O5	1.532 (3)	C9—C14	1.391 (6)
S1—C15	1.754 (6)	C10—C11	1.411 (6)
S1—C16	1.755 (6)	C11—H11	0.9300
S2A—O6	1.320 (6)	C11—C12	1.354 (7)
S2A—C17	1.717 (9)	C12—H12	0.9300
S2A—C18	1.724 (9)	C12—C13	1.378 (7)
S2B—O6	1.506 (6)	C13—C14	1.375 (6)
S2B—C17	1.611 (9)	C14—H14	0.9300
S2B—C18	1.641 (9)	C15—H15A	0.9600
O1—Fe1^i^	2.064 (2)	C15—H15B	0.9600
O1—C7	1.416 (4)	C15—H15C	0.9600
O2—C10	1.296 (5)	C16—H16A	0.9600
O3—N2	1.212 (6)	C16—H16B	0.9600
O4—N2	1.216 (6)	C16—H16C	0.9600
N1—C1	1.431 (5)	C17—H17A	0.9600
N1—C8	1.291 (5)	C17—H17B	0.9600
N2—C13	1.459 (7)	C17—H17C	0.9600
C1—C2	1.398 (6)	C17—H17D	0.9600
C1—C6	1.391 (5)	C17—H17E	0.9600
C2—C3	1.385 (5)	C17—H17F	0.9600
C2—C7	1.512 (5)	C18—H18A	0.9600
C3—H3	0.9300	C18—H18B	0.9600
C3—C4	1.367 (6)	C18—H18C	0.9600
C4—H4	0.9300	C18—H18D	0.9600
C4—C5	1.373 (7)	C18—H18E	0.9600
C5—H5	0.9300	C18—H18F	0.9600
C5—C6	1.370 (6)		
			
O1^i^—Fe1—Cl1	171.00 (8)	H7A—C7—H7B	107.4
O1—Fe1—Cl1	97.10 (8)	N1—C8—H8	117.2
O1—Fe1—O1^i^	75.23 (11)	N1—C8—C9	125.6 (4)
O1—Fe1—O5	99.53 (11)	C9—C8—H8	117.2
O1—Fe1—N1	82.16 (11)	C10—C9—C8	123.1 (4)
O1^i^—Fe1—N1	90.88 (11)	C14—C9—C8	117.2 (4)
O2—Fe1—Cl1	98.25 (9)	C14—C9—C10	119.5 (4)
O2—Fe1—O1	160.70 (12)	O2—C10—C9	123.0 (4)
O2—Fe1—O1^i^	90.25 (11)	O2—C10—C11	119.4 (4)
O2—Fe1—O5	91.92 (12)	C11—C10—C9	117.7 (4)
O2—Fe1—N1	85.49 (12)	C10—C11—H11	119.0
O5—Fe1—Cl1	90.66 (9)	C12—C11—C10	122.0 (5)
O5—Fe1—O1^i^	86.06 (10)	C12—C11—H11	119.0
O5—Fe1—N1	175.99 (12)	C11—C12—H12	120.3
N1—Fe1—Cl1	92.74 (9)	C11—C12—C13	119.4 (4)
O5—S1—C15	102.4 (2)	C13—C12—H12	120.3
O5—S1—C16	105.0 (2)	C12—C13—N2	120.4 (5)
C15—S1—C16	99.3 (4)	C14—C13—N2	118.0 (5)
O6—S2A—C17	112.6 (6)	C14—C13—C12	121.5 (5)
O6—S2A—C18	115.6 (5)	C9—C14—H14	120.0
C17—S2A—C18	97.6 (4)	C13—C14—C9	119.9 (4)
O6—S2B—C17	108.9 (4)	C13—C14—H14	120.0
O6—S2B—C18	110.4 (5)	S1—C15—H15A	109.5
C17—S2B—C18	105.5 (6)	S1—C15—H15B	109.5
Fe1—O1—Fe1^i^	104.77 (11)	S1—C15—H15C	109.5
C7—O1—Fe1	122.3 (2)	H15A—C15—H15B	109.5
C7—O1—Fe1^i^	125.6 (2)	H15A—C15—H15C	109.5
C10—O2—Fe1	124.5 (2)	H15B—C15—H15C	109.5
S1—O5—Fe1	122.83 (16)	S1—C16—H16A	109.5
C1—N1—Fe1	122.7 (3)	S1—C16—H16B	109.5
C8—N1—Fe1	118.0 (3)	S1—C16—H16C	109.5
C8—N1—C1	117.8 (3)	H16A—C16—H16B	109.5
O3—N2—O4	121.5 (5)	H16A—C16—H16C	109.5
O3—N2—C13	120.5 (5)	H16B—C16—H16C	109.5
O4—N2—C13	117.9 (5)	S2A—C17—H17A	109.5
C2—C1—N1	121.4 (3)	S2A—C17—H17B	109.5
C6—C1—N1	119.5 (4)	S2A—C17—H17C	109.5
C6—C1—C2	119.1 (4)	S2B—C17—H17D	109.5
C1—C2—C7	125.7 (3)	S2B—C17—H17E	109.5
C3—C2—C1	117.6 (4)	S2B—C17—H17F	109.5
C3—C2—C7	116.7 (4)	H17A—C17—H17B	109.5
C2—C3—H3	118.7	H17A—C17—H17C	109.5
C4—C3—C2	122.6 (4)	H17B—C17—H17C	109.5
C4—C3—H3	118.7	H17D—C17—H17E	109.5
C3—C4—H4	120.1	H17D—C17—H17F	109.5
C3—C4—C5	119.8 (4)	H17E—C17—H17F	109.5
C5—C4—H4	120.1	S2A—C18—H18A	109.5
C4—C5—H5	120.5	S2A—C18—H18B	109.5
C6—C5—C4	118.9 (4)	S2A—C18—H18C	109.5
C6—C5—H5	120.5	S2B—C18—H18D	109.5
C1—C6—H6	119.1	S2B—C18—H18E	109.5
C5—C6—C1	121.9 (4)	S2B—C18—H18F	109.5
C5—C6—H6	119.1	H18A—C18—H18B	109.5
O1—C7—C2	116.1 (3)	H18A—C18—H18C	109.5
O1—C7—H7A	108.3	H18B—C18—H18C	109.5
O1—C7—H7B	108.3	H18D—C18—H18E	109.5
C2—C7—H7A	108.3	H18D—C18—H18F	109.5
C2—C7—H7B	108.3	H18E—C18—H18F	109.5

Symmetry code: (i) −*x*+1, −*y*+1, −*z*.
